# Catecholamine-Stimulated Growth of *Aeromonas hydrophila* Requires the TonB2 Energy Transduction System but Is Independent of the Amonabactin Siderophore

**DOI:** 10.3389/fcimb.2016.00183

**Published:** 2016-12-12

**Authors:** Yuhao Dong, Jin Liu, Maoda Pang, Hechao Du, Nannan Wang, Furqan Awan, Chengping Lu, Yongjie Liu

**Affiliations:** ^1^Department of Preventive Veterinary, College of Veterinary Medicine, Nanjing Agricultural UniversityNanjing, China; ^2^Key Lab of Food Quality and Safety of Jiangsu Province-State Key Laboratory Breeding Base, Institute of Food Safety, Jiangsu Academy of Agricultural SciencesNanjing, China

**Keywords:** *Aeromonas hydrophila*, stress, catecholamine, TonB2 energy transduction system, amonabactin

## Abstract

The growth-stimulating effects of catecholamine stress hormones have been demonstrated in many pathogens. However, catecholamine-induced growth and its underlying mechanisms remain poorly understood in *Aeromonas hydrophila*. The present study sought to demonstrate that norepinephrine (NE), epinephrine (Epi), dopamine (Dopa), and L-dopa stimulate the growth of *A. hydrophila* in iron-restricted media containing serum. NE exhibited the strongest growth stimulation, which could be blocked by adrenergic antagonists. Furthermore, it was demonstrated that NE could sequester iron from transferrin, thereby providing a more accessible iron source for utilization by *A. hydrophila*. The deletion of the *amoA* gene associated with amonabactin synthesis revealed that the amonabactin siderophore is not required for NE-stimulated growth. However, the deletion of the TonB2 energy transduction system resulted in the loss of growth promotion by NE, indicating that a specific TonB-dependent outer membrane receptor might be involved in the transport of iron from transferrin. Collectively, our data show that catecholamine sensing promotes the growth of *A. hydrophila* in a manner that is dependent on the TonB2 energy transduction system.

## Introduction

*Aeromonas hydrophila* inhabits various aquatic environments and is responsible for motile Aeromonad septicemia (MAS), leading to large economic losses in the global aquaculture industry (Galindo et al., 2006). This bacterium also causes intestinal and extraintestinal infections in humans and other animals (Parker and Shaw, 2011). As well as being a beneficial symbiont, this bacterium often resides in the host without causing harm. However, once normal host defenses are compromised, *A. hydrophila* takes advantage of this opportunity to inflict damage to the host (Parker and Shaw, 2011). Thus, the stress status of a host may determine the outcome of an infection.

Stress is unavoidable in the aquaculture environment. The accumulation of *Vibrio splendidus* increased in juvenile oysters after mechanical stress, such as shaking (Lacoste et al., 2001). The exposure of fish to common stressors such as handling increased incidence of disease (Van Weerd and Komen, 1998). Peters et al. (1988) investigated the response of rainbow trout to the simultaneous exposure to stress and *A. hydrophila*, and found that this bacterium spread to more organs and was in greater number in the stressed fish compared to the unstressed fish. The mechanism of this phenomenon is unknown. One potential explanation is that stress-associated hormones may be released under stress, and these hormones depress the function of the immune system and exert deleterious effects on the immune response of the host (Freestone and Lyte, 2008). In addition, studies have shown that microbes can recognize and respond to neurohormone signals to modulate bacterial growth and virulence-associated gene expression (Burton et al., 2002; Sperandio et al., 2003). Norepinephrine (NE) has been reported to increase motility, chemotaxis, and the production of shiga toxins in *Escherichia coli* O157:H7 (Lyte et al., 1996; Bansal et al., 2007). Furthermore, NE increased both the cellular cytotoxicity and enterotoxicity of *Vibrio parahaemolyticus* and up-regulated the transcription of type III secretion system-1 genes (Nakano et al., 2007). Dopamine (Dopa) and epinephrine (Epi) have been found to increase biofilm formation in *Streptococcus pneumonia* (Sandrini et al., 2014). These observations indicate that stress hormones can regulate the behavior of pathogens. To date, information on the link between stress hormones and *A. hydrophila* remains limited. Only one study has reported that the treatment of *A. hydrophila* cultures with NE resulted in dramatic increases in bacterial growth (Kinney et al., 1999), but the mechanism underlying this has not been investigated.

It has previously been reported that growth promotion by stress hormones might be attributable to increased iron accessibility in bacterial cells (Freestone et al., 2003). And siderophore synthesis and uptake systems are key elements in the mechanism by which stress hormones induce growth (Burton et al., 2002; Freestone et al., 2003). Iron is an essential element for almost all living bacteria. Because of the low bioavailability of iron in the environment, bacteria have developed specific uptake strategies. *A. hydrophila* has been shown to possess multiple systems for the sequestration of host iron, including heme-bound iron transport (Maltz et al., 2015), the utilization of enterobactin siderophores produced by enterobacteriaceae (Funahashi et al., 2013), and the secretion of amonabactin (Barghouthi et al., 1989). After the ferri-siderophores and other iron sources specifically bind to outer membrane receptors, an energy generation system for transport is required (Postle and Larsen, 2007). During this process, the transmembrane transporter activity and ATP synthase has been demonstrated to increase significantly in abundance to enhance iron transport and maintain cellular iron homeostasis in *A. hydrophila* (Yao et al., 2016). Nevertheless, it remains unclear how *Aeromonas* species keep the iron homeostasis and its regulation under stress conditions.

The majority of our knowledge regarding bacteria-catecholamine interactions originates from studies of mammalian pathogens, and our understanding of the interactions between aquatic bacterial pathogens and catecholamine is relatively lacking. In the current study, we aimed to investigate the impact of NE and its related compounds on the growth of *A. hydrophila* NJ-35, an isolate from a diseased cyprinoid fish, in iron-restricted media, and to determine whether amonabactin is required for NE-mediated *A. hydrophila* growth promotion or whether other NE response mechanisms are present.

## Materials and methods

### Ethics statement

Experiments involving live animals were carried out according to animal welfare standards and were approved by the Ethical Committee for Animal Experiments of Nanjing Agricultural University, China. All animal experiments complied with the guidelines of the Animal Welfare Council of China.

### Bacterial strains and growth media

The bacterial strains and plasmids used in this study are listed in Table 1. *A. hydrophila* NJ-35, which belongs to the ST251 clonal group, was isolated from dead cultured cyprinoid fish in the Jiangsu province of China in 2010 (Pang et al., 2015).

**Table 1:** **Bacterial strains and plasmids used in this study**.

**Strain or plasmid**	**Description^a^**	**Source or reference**
**STRAINS**		
NJ-35	Wilde-type, isolated from diseased crucian carp, in China	Collected in our laboratory
SM10	*E. coli* strain, λ*pir*^+^, Kan^*r*^	Melton-Witt et al., 2012
Δ*amoA*	*amoA* deletion mutant from NJ-35	This study
Δ*tonB1*	*tonB1* deletion mutant from NJ-35	This study
Δ*tonB2-1*	*tonB2-1* deletion mutant from NJ-35	This study
Δ*tonB2-2*	*tonB2-2* deletion mutant from NJ-35	This study
Δ*tonB3*	*tonB3* deletion mutant from NJ-35	This study
**PLASMID**		
pYAK1	R6K-ori suicide vector, SacB^+^, Cm^r^	Abolghait, 2013
pYAK1-*amoA*	pYAK1 carrying the flanking sequence of *amoA*, Cm^r^	This study
pYAK1-*tonB1*	pYAK1 carrying the flanking sequence of *tonB1*, Cm^r^	This study
pYAK1-*tonB2-1*	pYAK1 carrying the flanking sequence of *tonB2-1*, Cm^r^	This study
pYAK1-*tonB2-2*	pYAK1 carrying the flanking sequence of *tonB2-2*, Cm^r^	This study
pYAK1-*tonB3*	pYAK1 carrying the flanking sequence of *tonB3*, Cm^r^	This study
pMMB207	Low-copy-number vector, Cm^r^	Morales et al., 1991
pMMB-*amoA*	Plasmid pMMB207 carrying the complete ORF of *amoA*	This study
pMMB-*tonB2-1*	Plasmid pMMB207 carrying the complete ORF of *tonB2-1*	This study
pMMB-*tonB2-2*	Plasmid pMMB207 carrying the complete ORF of *tonB2-2*	This study

a *Cm^r^, chloramphenicol resistant; Kan^r^, kanamycin resistant*.

*A. hydrophila* and *E. coli* strains were grown at 28 and 37°C, respectively, in Luria-Bertani broth (LB) or on LB agar plates. As necessary, antibiotics were used at the following concentrations: chloramphenicol (Cm) (Sigma, St. Louis, USA), 34 μg/ml for *A. hydrophila*; ampicillin (Amp) (Sigma), 100 μg/ml for *E. coli*. Serum-SAPI medium was used to assay growth promotion (Kinney et al., 2000), with some modifications. Briefly, serum-SAPI medium containing 6.25 mM ammonium nitrate, 3.35 mM potassium chloride 2.77 mM dextrose, 1.84 mM monobasic potassium phosphate and 1.01 mM magnesium sulfate was adjusted to pH 7.4 and supplemented with 10 mM HEPES buffer and 10% fetal bovine serum. The apo-form of bovine transferrin (ATF), the holo-form of bovine transferrin (HTF), chlorpromazine, phentolamine hydrochloride, and propranolol were purchased from Sigma-Aldrich (St. Louis, MO, USA). Norepinephrine (bitartrate salt), epinephrine (bitartrate salt), dopamine hydrochloride, L-dopa, tyrosine, and tyramine were purchased from Aladdin (Shanghai, China). All reagents were freshly prepared before each experiment and filter-sterilized using 0.22-μm (pore-size) membrane filters.

### Growth assays

For bacterial growth assays, *A. hydrophila* NJ-35 was grown overnight in LB medium at 28°C. Cells were pelleted by centrifugation at 5000 × g for 10 min, washed and resuspended in phosphate-buffered saline (PBS). An initial inoculum of ~10^2^ colony-forming units (CFU) ml^−1^ was diluted into serum-SAPI medium to obtain the strongest possible growth stimulation. In our pre-experiment, catecholamine hormones were confirmed to have no effect on *A. hydrophila* growth in serum-SAPI medium when the initial inoculum densities were >10^3^ CFU ml^−1^ (data not shown). Individual hormones were employed for experimental cultures at a final concentration of 100 μM, which was selected based on a preliminary study (Figure S1, take NE as an example). Additionally, a catecholamine receptor antagonist at a concentration of 400 mM was added to detect whether it exerted effects on catecholamine-induced growth (Freestone et al., 2007).

To investigate the role of transferrin in NE-induced growth, the serum in SAPI medium was replaced with either 39 μM (3 mg/ml) ATF or HTF. This concentration was chosen on the basis of previous reports that transferrin concentration in mammal serum is in the range 1–3.6 mg/ml (Burton et al., 2002; Kasvosve and Delanghe, 2002) and our preliminary dose-response studies, indicating that 3 mg/ml is the minimum concentration of the transferrin for the optimum growth of *A. hydrophila* (Figure S2). The initial concentration of bacteria was 1 × 10^3^ CFU/ml, higher than the concentration used in serum-SAPI medium, since there was no growth of *A. hydrophila* observed with the inoculums densities of <1 × 10^3^ CFU/ml. The cultures were grown in 1.5-ml EP tubes at 28°C for 20 h, and 100-μl aliquots of bacteria were removed from each tube every 2 h and plated on LB and the specific growth rate was calculated described previously (Lindqvist and Barmark, 2014). Each experiment was repeated four times.

### Biofilm assay

A biofilm formation assay were performed utilizing the crystal violet staining method described previously (Stepanovic et al., 2000), with some modification. *A. hydrophila* NJ-35 was grown overnight in LB broth at 28°C, bacteria were washed three times with PBS and diluted to an optical density of 0.01 at 600 nm (OD_600_) in serum-containing SAPI medium supplemented with 100 μM individual hormones, and 200-μl aliquots of suspension were dispensed into 96-well polystyrene plates. Then, the plates were incubated for 18 h at 28°C without agitation. Next, the contents of the wells were poured off, and wells were washed three times with 300 μl of sterile PBS. Adherent bacterial cells were fixed with 200 μl of methanol for 15 min, and then the methanol was removed. After drying for 15 min, 200 μl of crystal violet (1%wt/vol) per well was added, and staining was carried out for 10 min. Then, the wells were washed with ddH_2_O five times to remove unbound dye, and the plates were air-dried. The formed biofilms were solubilized in absolute ethanol, and the optical density was measured at OD_595_. Wells that did not contain bacteria served as a negative control.

### *In vitro* adhesion assays

HEp-2 cells were grown in Dulbecco's modified Eagle's medium (DMEM; Gibco, New York, USA) containing 10% fetal bovine serum (FBS; Gibco, New York, USA) in 24-well tissue culture plates to a final density of 5 × 10^5^ cells/well. The monolayers were washed with sterile 10 mM PBS to remove unattached cells, and then 400 μl of MEM without phenol red was added. *A. hydrophila* NJ-35 was grown in serum-SAPI medium in the presence or absence of 100 μM hormones for 18 h at 28°C under a microaerobic atmosphere, then harvested by centrifugation at 6000 × g for 10 min. The pellet was washed three times with PBS to remove hormones. The bacteria were seeded into each well at a multiplicity of infection (MOI) of 1:1. The plates were centrifuged at 600 × g for 10 min and incubated in a 5% CO_2_ humidified incubator for 2 h at 37°C. Subsequently, the cells were washed five times with PBS and lysed by adding 0.02% Triton X-100. Bacterial numbers were enumerated via serial dilution and plating on LB agar plates (Tenenbaum et al., 2005). All assays were performed with four replicates.

### Experimental infection of mice

Six- to eight-week-old female ICR mice were purchased from the Experimental Animal Center of Yangzhou University and housed under specific-pathogen-free conditions. Mice were fed intragastrically with 1 mg of NE in 400 μl of PBS 12 h before bacterial challenge. Mice administered PBS intragastrically served as controls. The dose of NE used in this study was chosen on the basis of a preliminary study, in which the starting dose was screened from the fixed levels of 0.5, 1, and 2 mg expected to show the altered bacterial loads but no obvious clinical signs or pathologic changes in animals. Then, the mice were infected with a predetermined dose of 1 × 10^8^ CFU of *A. hydrophila* NJ-35 per animal in a 200-μl suspension by intragastric administration. At 6 h postinfection, mice were euthanized. Lungs and spleens were aseptically removed, homogenized, and diluted in PBS. Bacterial loads in tissues were counted via serial dilution of the suspensions and plating on LB agar plates.

### Effects of NE on iron release from transferrin

To test the ability of NE to acquire iron from Tf, 13 μM (1 mg/ml) HTF was prepared in SAPI medium buffer supplemented with 100 mM Tris-HCl buffer at pH 7.5, and experimental cultures were supplemented with 100 μM NE and incubated at 37°C for 12 h, while control cultures contained an equivalent volume of water only. Samples were analyzed by electrophoresis in 6% polyacrylamide gels containing 6 M urea in a BioRad Protean II vertical mini gel system as previously described with certain modifications (Wolz et al., 1994). The gels were prepared as follows: 4.5 g of urea was added into 2.7 ml of acrylamide (3.3% C–30% T) and 2.5 ml TBE buffer concentrated 5 times, and the volume was adjusted to a total of 12.5 ml with deionized water. The gels were polymerized with the addition of 100 μl of 10% ammonium persulfate and 6 μl of TEMED. Electrophoresis was performed at 200 V for 6 h. The gels were stained with Coomassie Brilliant Blue R-250 (Bio-Rad).

### Construction of gene deletion mutants

*amoA* gene inactivation was carried out via homologous recombination using the suicide plasmid pYAK1 as previously described (Abolghait, 2013). First, the left and right arms of the *amoA* gene were PCR-amplified from the chromosomal DNA of *A. hydrophila* NJ-35 using two sets of primer pairs, AmoA-1/AmoA-2 and AmoA-3/AmoA-4 (Table 2), and the arms were then used as templates to generate fusion fragments with the primer pair AmoA-1/AmoA-4. These fusion fragments were cloned into the pYAK1 suicide plasmid with the restriction enzyme *Bam*HI. The recombinant plasmid pYAK1::*amoA* was transformed into *E. coli* SM10 competent cells (Melton-Witt et al., 2012). The donor strain *E. coli* SM10-pYAK1 (Cm resistant, Cm^r^) and the recipient strain *A. hydrophila* NJ-35 (Amp resistant, Amp^r^) were cultured in LB broth without antibiotics until log phase was reached. Cells were mixed at a ratio of two-to-one vol/vol in medium, spotted on a nylon filter on an LB plate and conjugated for 12 h at 28°C. Cells were recovered and washed three times with PBS. LB agar plates containing 100 μg/ml Amp and 34 μg/ml Cm were used to select for recombinant plasmid integration into the chromosome. Then, colonies (Amp^r^ and Cm^r^) were chosen and inoculated in LB broth supplemented with 20% sucrose to induce a second crossover event (Abolghait, 2013). The double-crossover Δ*amoA* mutant strain was confirmed by sequencing the deleted region and flanking DNA in the mutated strains. Further, quantitative real-time reverse transcription-PCR (qRT-PCR) was used to measure the transcriptional levels of upstream or downstream genes of the deletion regions, demonstrating that no polar mutation occurred due to the knock-out of *amoA*. Using the same approach, additional deletion mutants including Δ*tonB1*, Δ*tonB2-1*, Δ*tonB2-2*, and Δ*tonB3* were also constructed.

**Table 2:** **Primers used in this study**.

**Primer**	**Sequence (5′→ 3′)^a^**	**Function**
AmoA-1	CAGGTCGACTCTAGAGGATCC CTTCGTTCTTGCGAC	Construction of *amoA* deletion mutant
AmoA-2	ACTGGCTCAT GTTACACCCTCAAATATGATTC	
AmoA-3	AGGGTGTAAC ATGAGCCAGTCCAACCGC	
AmoA-4	GAGCTCGGTACCCGGGGATCC GGCAATCAGCGGGAAACA	
TonB1-1	CAGGTCGACTCTAGAGGATCCGCCTCTGTCTGGTTT	Construction of *tonB1* deletion mutant
TonB1-2	GAGACAAGTGACATGGATCCTGAAATCGC	
TonB1-3	GGATCCATGT CACTTGTCTCCCTTCCAG	
TonB1-4	GAGCTCGGTACCCGGGGATCCGCACGAACGGGTTATTT	
TonB2-1-1	CAGGTCGACTCTAGAGGATCC GTTTCATCTGTCCCTT	Construction of *tonB2-1* deletion mutant
TonB2-1-2	ACCGAAATGA CTATGTTGCGGATCTGGA	
TonB2-1-3	CGCAACATAG TCATTTCGGTGCCACC	
TonB2-1-4	GAGCTCGGTACCCGGGGATCC GCGGCTGCTCTACCTCAA	
TonB2-2-1	CAGGTCGACTCTAGAGGATCC CCAGTCCCAGGCTC	Construction of *tonB2-2* deletion mutant
TonB2-2-2	CCAGTCATGA ATGATGCTCGACATCTGG	
TonB2-2-3	CGAGCATCAT TCATGACTGGGCCTCCT	
TonB2-2-4	GAGCTCGGTACCCGGGGATCC AGCCTACAACCGCTACAT	
TonB3-1	CAGGTCGACTCTAGAGGATCC ATGGGATTGCCCTTG	Construction of *tonB3* deletion mutant
TonB3-2	GACTATTACAATGAAAGGAATCAAACTTGC	
TonB3-3	TTCCTTTCAT TGTAATAGTCCTTGTTTTCATAG	
TonB3-4	GAGCTCGGTACCCGGGGATCC CCAGACCCAGTTCTATCAG	
AmoA-C-F	CAGGAAACAGAATTCGAGCTCTCAGCTGCTCTTGCTCG	Construction of *amoA* complemented strain
AmoA-C-R	GGATCCCCGGGTACCGAGCTCTATCGCCTCCCAGACCA	
TonB2-1-C-F	CAGGAAACAGAATTCGAGCTCTTATGACTCCAGTTTGAATTTGA	Construction of *tonB2-1* complemented strain
TonB2-1-C-R	GGATCCCCGGGTACCGAGCTCGCAGGCCTATCAAATCGA	
TonB2-2-C-F	CAGGAAACAGAATTCGAGCTCTTACGGCTCCGGCTGG	Construction of *tonB2-2* complemented strain
TonB2-2-C-R	GGATCCCCGGGTACCGAGCTCCGCCGCCAGCCAAC	

a *Underlined sequences indicate restriction sites*.

### Construction of the complementation strain

The complementation of Δ*amoA* strain was constructed with shuttle plasmid pMMB207. The DNA fragments, including *amoA* gene and its putative promoter and terminator region, were amplified using the primer pair (AmoA-C-F/R) with restriction enzyme sites *Sac I*. Following digested and purified, the target gene was ligated into the pMMB207 vector. The recombinant plasmid pMMB207-*amoA* was first introduced into *E. coli* SM10 by chemical transformation, and then transformed into the mutant strain Δ*amoA* using bacterial conjugation, thus generating the complemented strain CΔ*amoA*. PCR amplification and sequencing were performed to verify the complementation strain. Using the same approach, the complemented strain CΔ*tonB2-1* and CΔ*tonB2-2* were also constructed.

### Siderophore CAS plate assays

Siderophore production was examined using chrome azurol S (CAS) plate (Schwyn and Neilands, 1987). Wild-type and Δ*amoA* mutant strains were incubated in LB medium overnight, and the cells were collected by centrifugation at 5000 × g for 10 min, then washed and resuspended in PBS. Cells were normalized to an OD_600_ of 1 in PBS. Ten microliters of suspension was spotted on a CAS agar plate. The CAS plates were incubated for 24 h at 28°C.

### Statistical analysis

Statistical analyses were performed using SPSS software (SPSS for Windows 16, SPSS Inc., Chicago, IL, USA). Multiple comparisons were performed using analysis of variance (ANOVA) followed by Bonferroni's post-test. The animal infection study analysis was performed using the nonparametric Mann–Whitney *U*-test. *P* < 0.05 was considered statistically significant.

## Results

### Effect of catecholamines on *A. hydrophila* growth

**Figure 1:**
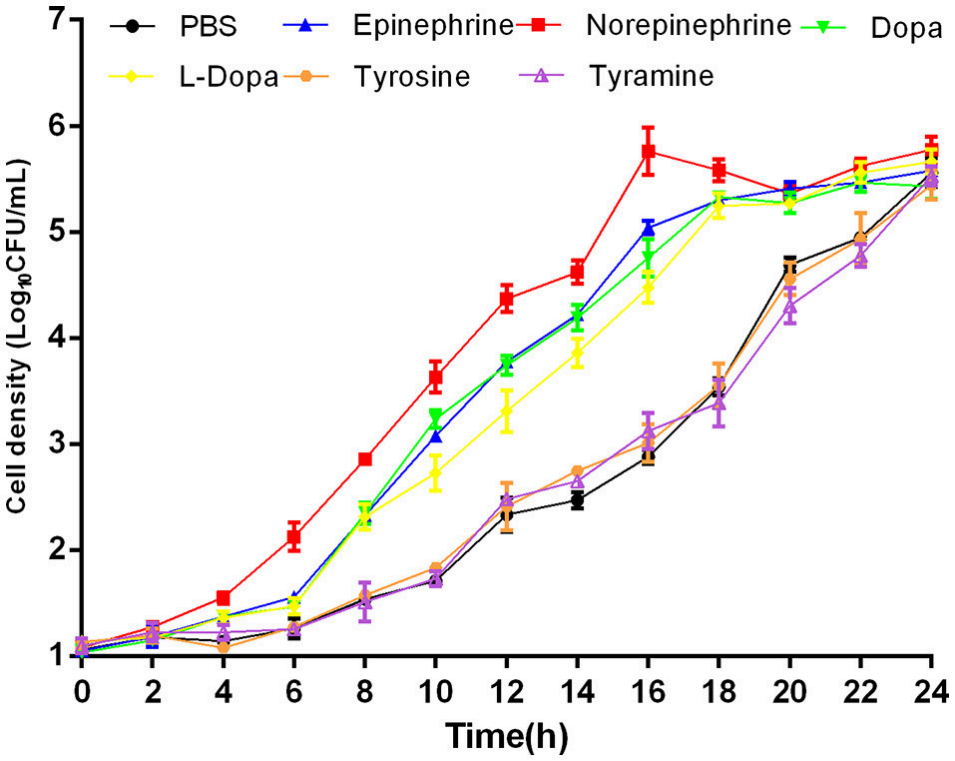
**Growth of *A. hydrophila* NJ-35 in the presence of NE and NE biosynthetic intermediates**. An initial inoculum of *A. hydrophila* at approximately 10^2^ CFU/ml was grown in serum-containing medium supplemented with individual hormones at a concentration of 100 μM at 28°C in a humid atmosphere containing 5% CO_2_. Results are shown as the means ± SEM from four independent replicates.

In this study, NE, Epi, Dopa, L-dopa, tyrosine, and tyramine were evaluated for *A. hydrophila* growth promotion. The data showed that in serum-SAPI minimul medium, NE, Epi, Dopa, and L-dopa significantly stimulated the growth of *A. hydrophila* (Figure 1), and resulted in a 1.81-, 1.72-, 1.75-, and 1.72-fold increase (*P* < 0.05) in the specific growth rate of *A. hydrophila*, respectively (0.58 ± 0.12, 0.55 ± 0.03, 0.56 ± 0.03, and 0.55 ± 0.15 h^−1^ in the presence of NE, Epi, Dopa, and L-dopa, respectively, compared to 0.32 ± 0.09 h^−1^ for the control). The growth of *A. hydrophila* in the presence of tyrosine and tyramine exhibited no increase compared to the control (0.33 ± 0.23 and 0.30 ± 0.29 h^−1^ in the presence of tyrosine and tyramine, respectively, compared to 0.32 ± 0.09 h^−1^ for the control).

**Figure 2:**
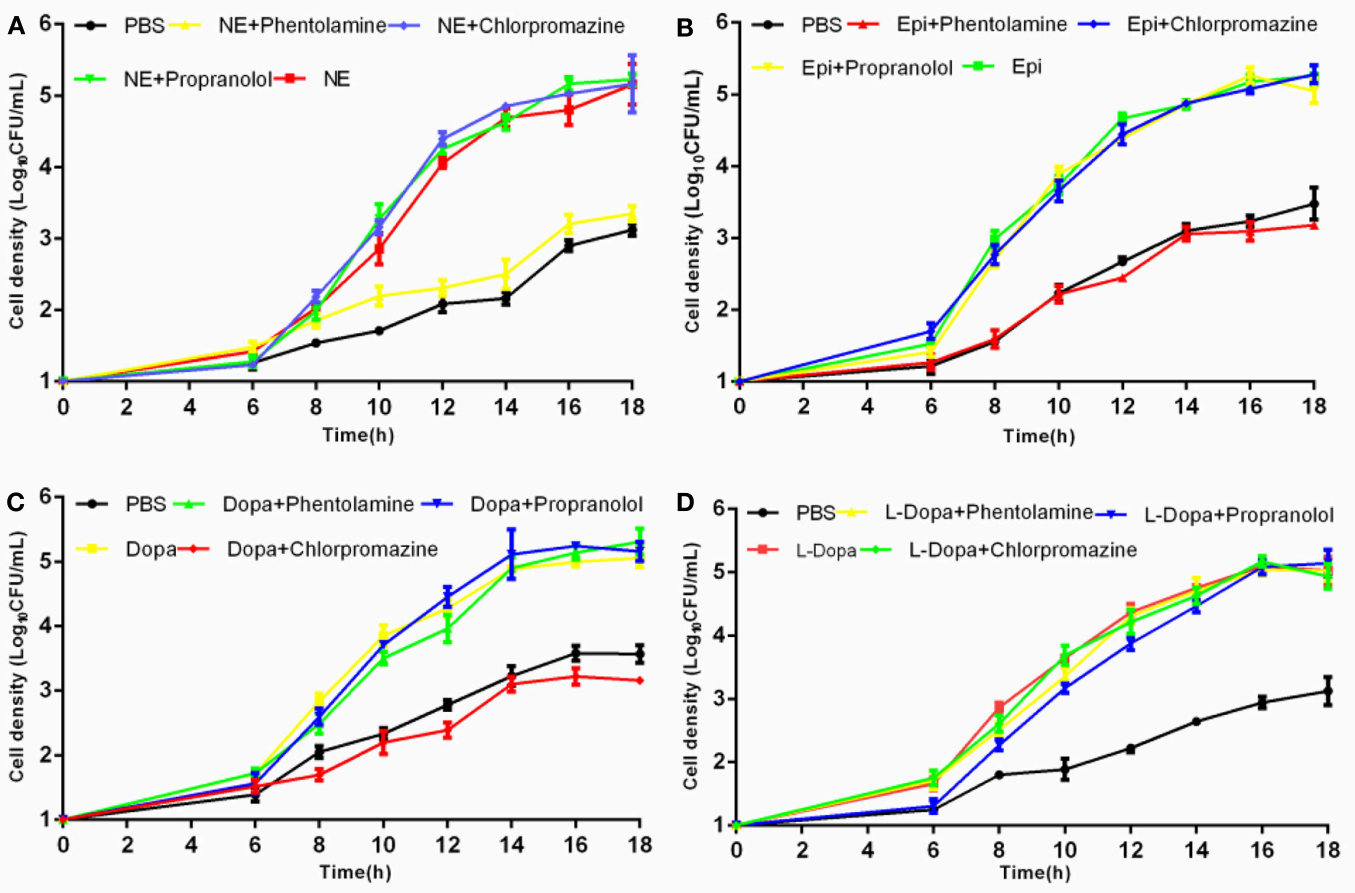
**Effects of receptor antagonists on hormone-induced growth responsiveness in *A*. *hydrophila* NJ-35**. Catecholamine receptor antagonists were added at a concentration of 400 mM to investigate the growth induced by NE **(A)**, Epi **(B)**, Dopa **(C)**, and L-dopa **(D)**. Results are shown as the means ± SEM from three independent replicates.

To evaluate whether growth promotion by catecholamines could be blocked, we performed growth inhibition tests employing adrenergic and dopaminergic-type receptor antagonists. The data demonstrated that the α-adrenergic antagonist phentolamine was able to inhibit growth induction by Epi and NE but could not block growth responses to Dopa and L-dopa. The dopaminergic receptor antagonist chlorpromazine was only able to inhibit growth induction by Dopa (Figure 2). Furthermore, the β-adrenergic antagonist propranolol had no effect on NE-, Epi-, Dopa-, or L-dopa-induced growth. Receptor antagonists did not affect the growth of *A. hydrophila* when used alone (data not shown). This indicates that growth inhibition is the result of specific antagonism of the bacterial response to stress hormones but does not result from antagonist toxicity. To exclude the possibility that the receptor antagonists directly bind NE, we examined the iron release from transferrin in the presence of NE- phentolamine mixture by denaturing urea-PAGE analysis (**Figure 7**). We found that the addition of receptor antagonists did not influence the iron removal from transferrin by NE, indicating that the antagonist effect was caused by a blockade of bacteria to NE.

### Effects of catecholamines on biofilm formation and bacterial adherence

**Figure 3:**
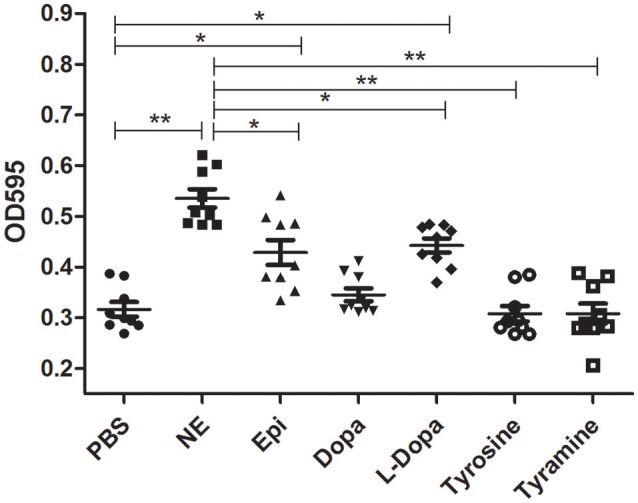
**Biofilm formation of *A. hydrophila* NJ-35 in the presence of NE and NE biosynthetic intermediates**. *A. hydrophila* NJ-35 was grown in serum-containing medium supplemented with individual hormones at a concentration of 100 μM. Crystal violet staining was performed to examine biofilm formation. ^*^P < 0.05 or ^**^P < 0.01.

Crystal violet staining assays were carried out to examine whether catecholamines impacted the formation of biofilms. The results showed that biofilm formation was increased after 16 h by the addition of NE, Epi, or L-dopa, and NE exerted the strongest effect on biofilm formation (Figure 3). Before performing this assay, we examined the growth of *A. hydrophila* at an initial inoculum of 5 × 10^6^ CFU/ml. No significant modification in growth was observed following exposure to the hormones used in this assay (data not shown). This result indicates that the enhancement of biofilm formation is not attributable to the effects of the hormones on growth rate.

**Figure 4:**
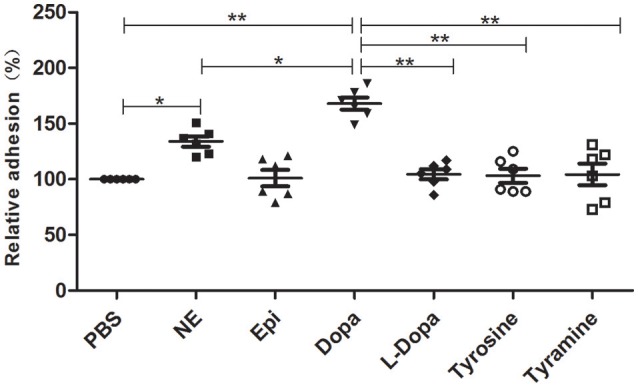
***A. hydrophila* NJ-35 adhesion to HEp-2 cells in the presence of NE and NE biosynthetic intermediates**. *A. hydrophila* NJ-35 was grown in serum-SAPI medium supplemented with individual hormones at a concentration of 100 μM for 18 h at 28°C and was then seeded into HEp-2 cells at an MOI of 1. Adherent bacterial numbers were counted by plating out the lysates of infected HEp-2 cells on LB agar. ^*^P < 0.05 or ^**^P < 0.01.

An *in vitro* adhesion assay showed that supplementation with NE and Dopa could significantly enhance *A. hydrophila* adhesion to HEp-2 cells (Figure 4). Other molecules used in this assay induced no changes in adherence. Our findings demonstrated that pathogenic phenotypes such as biofilm formation and adhesion to HEp-2 cells can be regulated by different stress hormones.

### NE enhances the systemic spread of *A. hydrophila in vivo*

**Figure 5:**
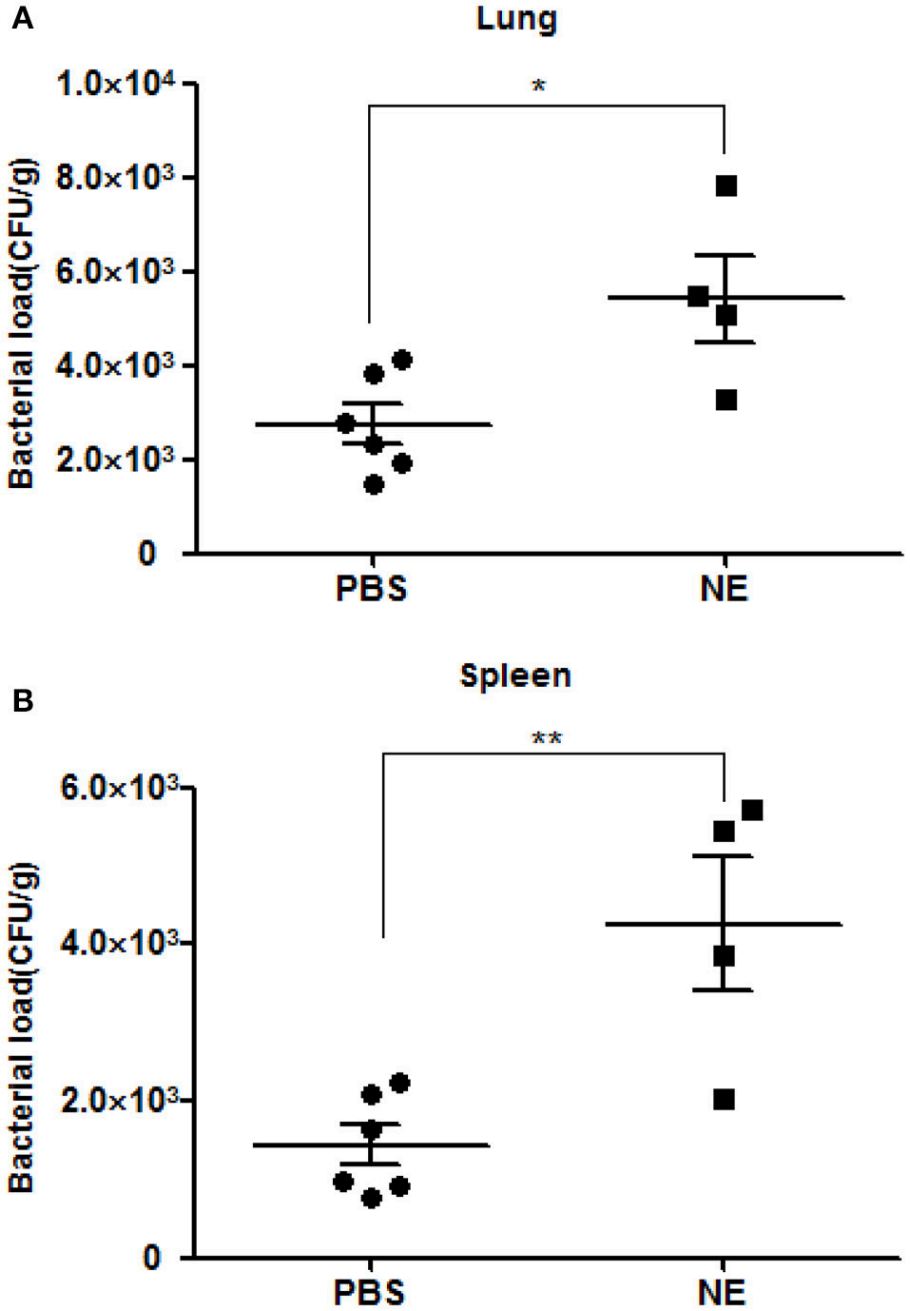
**Bacterial loads in mice with or without NE pre-treatment**. Bacterial loads in the spleen **(A)** and lung **(B)** are expressed as CFU/g of tissue. The experiment was repeated three times. ^*^P < 0.05 or ^**^P < 0.01.

To corroborate whether NE can affect the course of *A. hydrophila* proliferation *in vivo*, we performed an infection assay involving the artificial modulation of catecholamine levels in mice. Groups of six mice were intragastrically administered 0 or 1 mg of NE in 400 μl of PBS 12 h before infection with *A. hydrophila* NJ-35. Mice were sacrificed at 6 h postinfection to collect their spleens and lungs, and the bacterial loads were assessed in these tissues. The pre-treatment of mice with NE after challenge with *A. hydrophila* NJ-35 resulted in marked enhancements in bacterial colonization compared to non-pre-treated controls. As shown in Figure 5, approximate 3- and 2-fold increases in the spleen and lung, respectively, were observed compared to the controls. This result demonstrated that NE can increase the systemic spread of *A. hydrophila* NJ-35 and accelerate the course of infection.

### Involvement of Tf in growth stimulation by NE in SAPI medium

**Figure 6:**
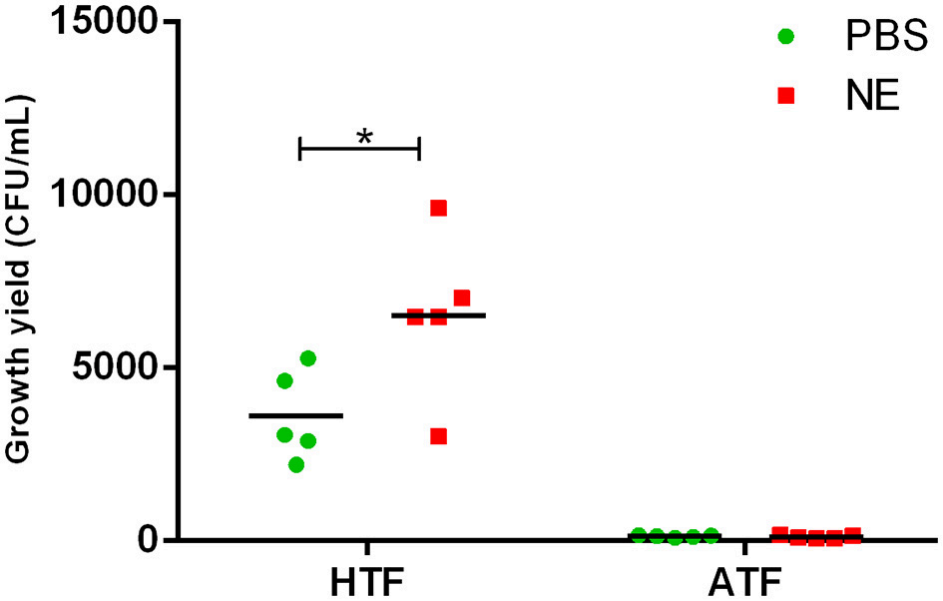
**Growth of *A. hydrophila* NJ-35 in SAPI medium containing apo-transferrin (ATF) or holo-transferrin (HTF) in the presence of NE**. The initial inoculum of *A. hydrophila* NJ-35 was 1 × 10^3^ CFU/ml, and the concentration of transferrin was 39 μM (3 mg/ml). Data are expressed as the means ± SEM of five independent replicates. ^*^P < 0.05.

**Figure 7:**
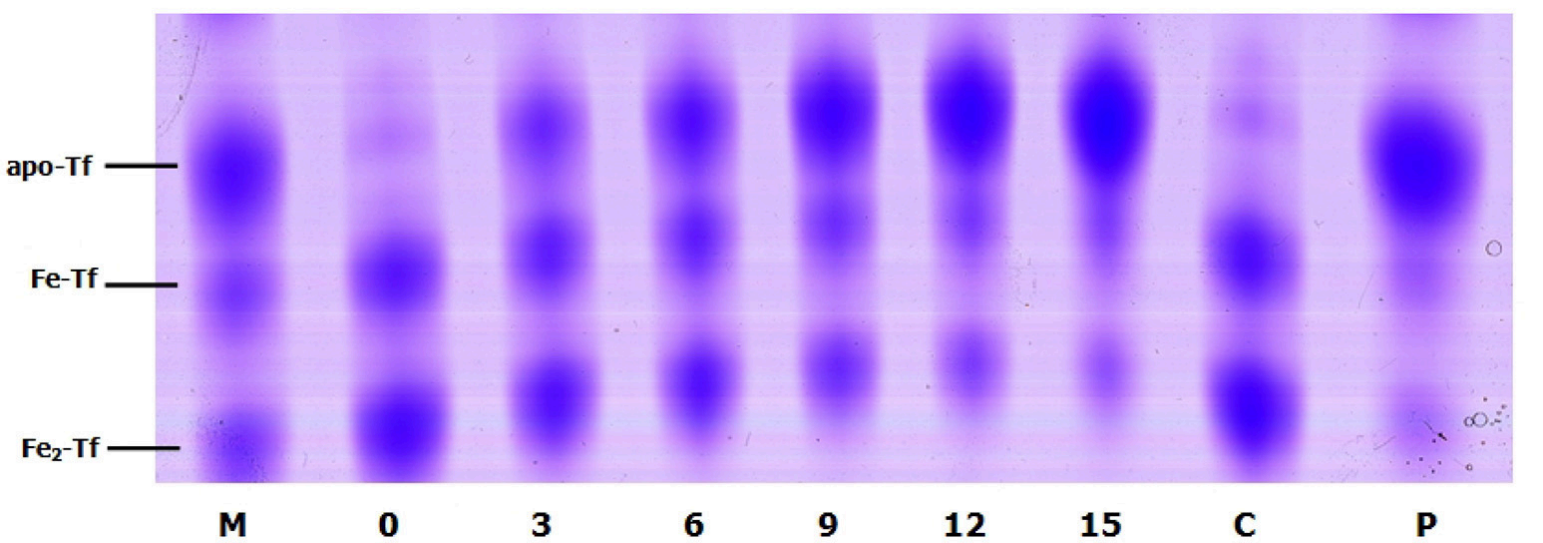
**Urea polyacrylamide gel electrophoresis demonstrating iron removal from Tf in the presence of NE over time**. *A. hydrophila* NJ-35 was inoculated in HTF-SAPI medium containing 100 mM Tris-HCl buffer at 37°C. The numbers below the lanes represent the number of hours of incubation with NE. Lane M contains the iron-free (apo-Tf), monoferric (Fe-Tf), and saturated (Fe_2_-Tf) isoforms as markers. Lane C shows Fe_2_-Tf incubated in the medium for 15 h without NE. Lane P shows Fe_2_-Tf incubated in the medium for 15 h with NE and phentolamine.

To evaluate the role of transferrin in the NE-induced growth stimulation of *A. hydrophila*, we performed a growth assay employing 39 μM transferrin instead of serum in SAPI medium. As expected, NE enhanced the growth of *A. hydrophila* in SAPI medium containing holotransferrin (Figure 6). This result is similar to the results that we obtained in the NE-mediated growth stimulation assay with the addition of serum into SAPI medium (Figure 1). In contrast, *A. hydrophila* growth did not significantly change in apotransferrin-containing medium in the presence of NE. These results indicate that holotransferrin can take the place of serum in NE-induced growth stimulation. Further, the urea-PAGE analysis of the Tf-NE complex showed that in the presence of NE, purified diferric Tf was gradually converted into the monoferric or apo forms of Tf (Figure 7).

### Stimulation of *A. hydrophila* growth is not mediated by amonabactin

**Figure 8:**
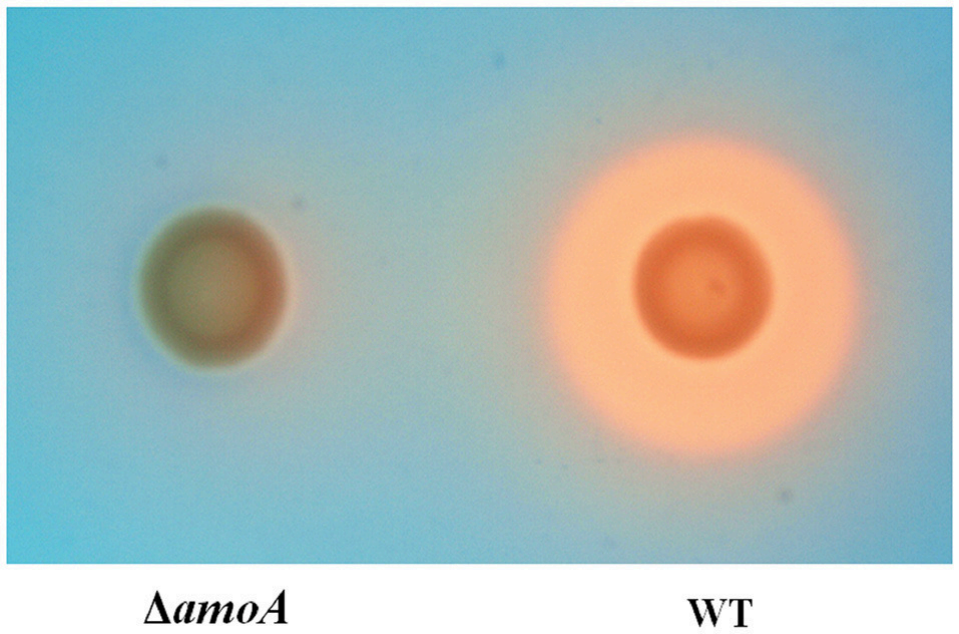
**Assessment of the ability of wild-type (WT) and Δ*amoA* mutant strains of *A. hydrophila* NJ-35 to produce amonabactin as determined by CAS plate assay**. The production of amonabactin is detected as an orange halo resulting from the removal of iron from the blue-colored CAS dye complex.

**Figure 9:**
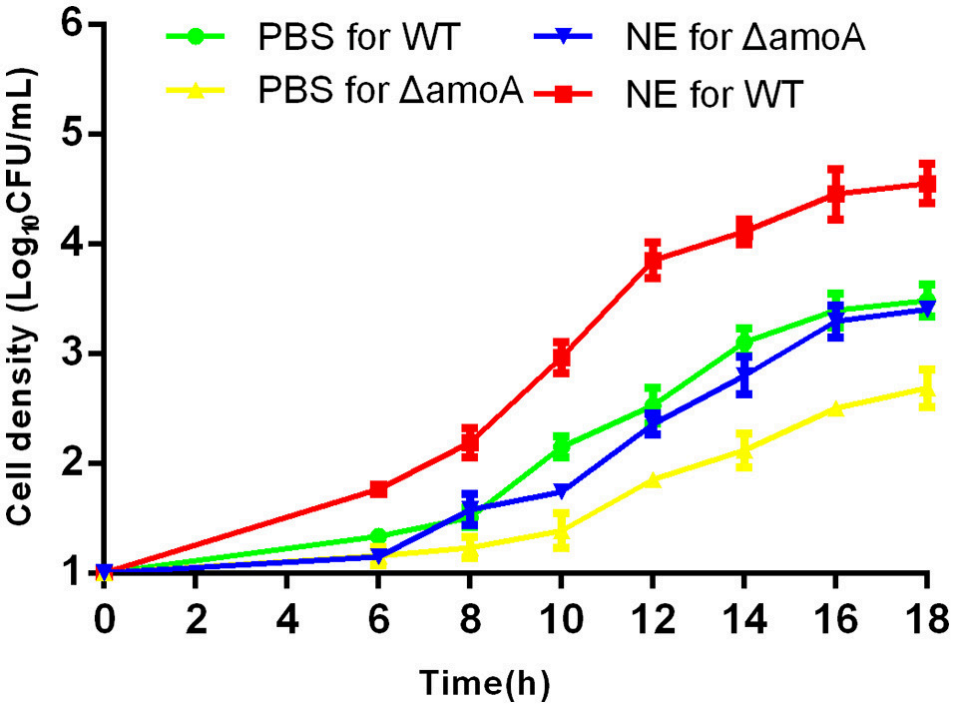
**Growth stimulation by NE is independent of amonabactin**. *A. hydrophila* NJ-35 wild-type (WT) and Δ*amoA* strains were grown in serum-supplemented SAPI medium in the presence or absence of NE. Results are shown as the means ± SEM from four independent replicates.

**Figure 10:**
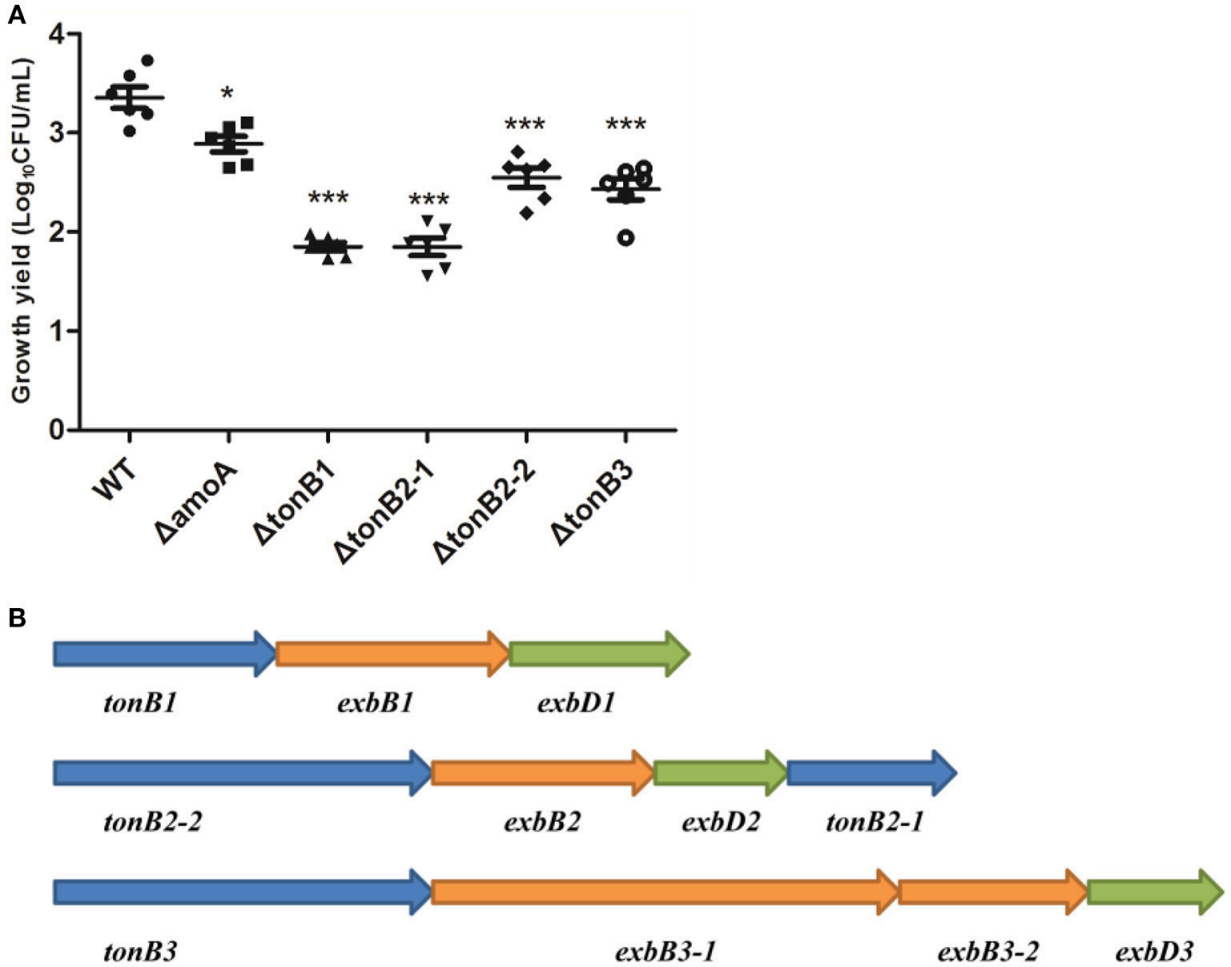
**Growth of the wild-type *A. hydrophila* strain, its derivative *amoA* and the *tonB* mutants in serum-supplemented medium (A)** and genetic organization of the three-TonB system gene clusters of *A. Hydrophila* NJ-35 **(B)**. The growth yield is expressed as Log_10_CFU/ml, and results are shown as the means ± SEM from six independent replicates. ^*^P < 0.05, or ^***^P < 0.001.

To determine whether NE-enhanced growth of *A. hydrophila* NJ-35 was mediated by amonabactin, we constructed an isogenic deletion mutant of *amoA*. The CAS plate assay showed that the Δ*amoA* mutant failed to produce amonabactin (Figure 8). The addition of 100 μM NE enhanced the growth of the Δ*amoA* mutant (Figure 9) and resulted in a 1.45-fold increase (*P* < 0.05) in the specific growth rate (0.32 ± 0.06 h^−1^ in the presence of NE, compared to 0.22 ± 0.03 h^−1^ for the untreated control), while the similar NE-mediated growth promotion was observed for the wild-type strain (0.45 ± 0.23 h^−1^ in the presence of NE, compared to 0.32 ± 0.20 h^−1^ for the untreated control). Moreover, compared with the wild-type strain, the Δ*amoA* mutant strain showed a significant growth defect in serum-SAPI medium (Figure 10A). The complementary strain, CΔ*amoA* could restore the production of amonabactin, and the CΔ*amoA* growth was almost restored to the wildtype level in the presence or absence of NE (data not shown). These data indicated that amonabactin plays a crucial role in growth in serum-based medium, whereas the ability of *A. hydrophila* to synthesize amonabactin is not essential for NE-dependent growth promotion in serum-supplemented medium.

### TonB2 contributes to NE-dependent growth promotion in *A. hydrophila*

**Figure 11:**
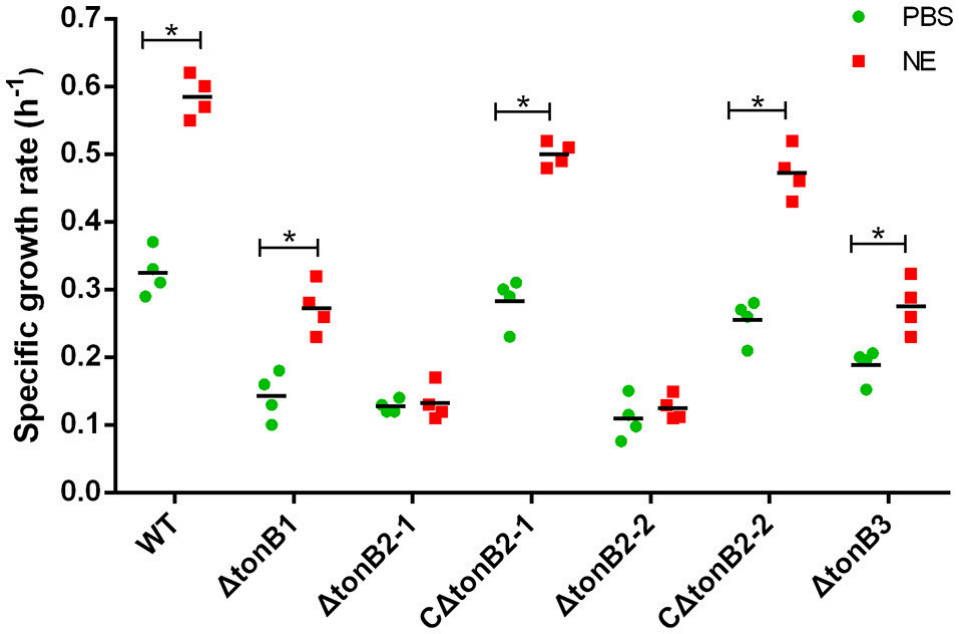
**TonB2-dependent growth stimulation by NE in serum-supplemented SAPI medium**. The specific growth rate calculated from the cell densities measured during exponential growth phase. The results are shown as the means ± SEM from four independent replicates. ^*^P < 0.05.

As the present study indicated that NE-enhanced *A. hydrophila* growth was attributable to increased iron availability in the bacterial cell, we determined whether this process required the TonB-dependent transport system. The TonB system is responsible for energizing transporters in the outer membrane (Postle and Larsen, 2007). A homology search of the genome sequence of *A. hydrophila* NJ-35 revealed the presence of three TonB systems, encoded by *tonB1-exbB1-exbD1, tonB2-2-exbB2-exbD2-tonB2-1*, and *tonB3-exbB3-exbB3-exbD3* (Figure 10B). To determine which TonB system is involved in the NE-mediated growth enhancement of *A. hydrophila*, we constructed *tonB1, tonB2-1, tonB2-2*, and *tonB3* single deletion mutants. The Δ*tonB2-1* and Δ*tonB2-2* strains demonstrated no responses to NE in serum-containing medium (0.13 ± 0.09 and 0.13 ± 0.04 h^−1^ in the presence of NE, respectively, compared to 0.12 ± 0.03 and 0.11 ± 0.05 h^−1^ for the untreated control), whereas the growth of the complemented strain was almost restored to the level of the wild-type strain in responses to NE (Figure 11). However, NE still enhanced the growth of both the Δ*tonB1* and Δ*tonB3* mutants (Figure 11) and resulted in a 2.0- and 1.5-fold increase (*P* < 0.05) in the specific growth rate, respectively (0.28 ± 0.07 and 0.28 ± 0.06 h^−1^ in the presence of NE, respectively, compared to 0.14 ± 0.03 and 0.19 ± 0.10 h^−1^ for the untreated control). In addition, the growth yields of all four mutants were markedly lower than that of the parental strain in serum-supplemented medium (Figure 10A). These results indicated that only the TonB2 system is necessary for NE-dependent growth stimulation in serum-containing medium. Furthermore, it is notable that all three TonB systems are required for the optimal growth of *A. hydrophila* in serum-containing medium.

## Discussion

In the recent years, microbial endocrinology has revealed that many microorganisms have evolved specific mechanisms to sense and respond to stress hormones. However, most investigations to date have concentrated on mammalian pathogens. Epinephrine, dopamine and norepinephrine are the predominant stress hormones that are released from the chromaffin tissue located in the head kidney (Perry and Capaldo, 2011) as well as from adrenergic nerve endings in fish (Gamperl et al., 1994). To determine the effect of the catecholamine stress hormones on *A. hydrophila* growth, in this study, we used a minimal medium (SAPI) supplemented with serum to simulate *in vivo* iron-restricted environment. In considering the catecholamine concentrations used in the present study, it should be emphasized that micromolar concentrations are meant to mimic the concentrations in the host. However, because catecholamines levels can change according to stress level, tissue location and circadian rhythm, the actual catecholamine concentrations inside tissues and blood are difficult to accurately determine (Li et al., 2012). For example, higher concentrations can be found in innervated tissues such as the gut, and the concentration of NE can be up to 10 mM in neuronal synapses (Lyte, 2004). The concentration of catecholamines used in this study (100 μM) is, therefore, biologically relevant. Similar concentration range has been used in a previous study, which has investigated *A. hydrophila* responses to catecholamines (Kinney et al., 1999).

Our study demonstrated that NE, Epi, Dopa, and L-dopa could significantly increase *A. hydrophila* growth in SAPI-serum medium, whereas tyrosine and tyramine did not significantly alter the bacterial growth. These results suggest that *A. hydrophila* has evolved catecholamine response systems for the specific hormones. Further investigation demonstrated that the induced growth of *A. hydrophila* by Epi and NE could be blocked by α- but not β-receptor antagonists; however, the α-receptor antagonist did not neutralize induction by Dopa. In contrast, the dopaminergic receptor antagonist blocked growth induction by Dopa but did not demonstrate any effects on other catecholamines with the ability to stimulate bacterial growth. This may reflect the fact that bacterial receptor systems for catecholamine possess a degree of specificity, similar to mammalian catecholamine receptors (Yang et al., 2014). In terms of bacterial catecholamine receptors, there is so far no genomic evidence for the existence of a classical adrenergic receptor motif in bacterial species. However, Clarke et al. (2006) reported that NE was able to bind to the *E. coli* O157:H7 two-component regulator sensor kinase QseC, leading to the hypothesis that QseC is the bacterial catecholamine receptor. Later on, there are increasing reports of alternative receptors which participate in bacterial responsiveness to adrenaline or noradrenaline, such as BasSR and CpxAR two-component signal transduction systems (Humphreys et al., 2004; Marchal et al., 2004). In this paper, the evidence from the antagonist experiments indicates the presence of specific recognition systems for NE, Epi and Dop that are essential for induction of bacterial growth. However, unequivocal evidence for the existence of bacterial α-adrenergic and dopaminergic receptors in *A. hydrophila* will require further investigation.

In this study, we showed that catecholamines can increase biofilm formation and adhesion, which are crucial for *A. hydrophila* infections. Norepinephrine exerted the strongest impact on biofilm formation, but the effects of Dopa on bacterial adhesion were greater than those of norepinephrine. These discrepancies may be attributable to the possibility that there is more than one system to respond to stress hormones. In *E. coli* O157:H7, neuroendocrine hormones are sensed by the QseBC and QseEF two component systems (Rasko et al., 2008), which are involved in regulating motility and the expression of the enterocyte effacement (LEE) gene locus, respectively (Reading et al., 2007). In this regard, it may be interesting to further evaluate which systems contribute to catecholamine hormone sensing and the regulation of stress-associated gene expression in *A. hydrophila*.

Additionally, *in vivo* experiment showed that NE administration increased bacterial loads in the spleen and lung of mice at 6 h post-infection. It is not clear which mechanism encourages the colonization in tissues by *A. hydrophila*. However, the addition of NE resulted in enhanced bacterial adherence and biofilm production *in vitro*, which implies that NE increased fitness for transmission of *A. hydrophila* in mice. Furthermore, whether NE impairs host immune system is not known at this time, but is a future investigative objective of our laboratories.

Intriguingly, we found that all growth-stimulatory hormones employed in this assay contained a 3,4-dihydroxybenzoyl moiety. A previous study showed that the 3,4-dihydroxybenzoyl (catechol) structure is an essential element for the removal of iron from transferrin (Eisenhofer et al., 1996). Furthermore, some metabolites of catecholamine hormones that contain the catechol moiety, such as dihydroxyphenyl glycol, and dihydroxymandelic acid, have been demonstrated to exert growth-promoting effects (Nakano et al., 2007). Thus, we speculate that hormone-mediated growth promotion may be involved in facilitating the utilization of iron by bacteria.

In the host, the concentration of freely available iron is very low in the circulation. Ferric iron is generally sequestered by high-affinity iron-chelating proteins such as transferrin in the plasma (Schade and Caroline, 1946). Here, we investigated the role of transferrin in the NE-induced *A. hydrophila* growth. Similar to the effect of serum, the addition of transferrin to SAPI medium enhanced the growth of *A. hydrophila*. Further, we demonstrated that iron could be released from transferrin in the presence of NE, as determined by denaturing urea-PAGE analysis. The data indicated that NE has the ability to remove iron from ferric transferrin in *A. hydrophila*.

For many pathogens, the acquisition of iron generally involves the production and extraction of siderophores (Meyer et al., 1996). Siderophores can capture iron from transferrin or inaccessible ferric iron found in the environment and subsequently deliver it into the bacterial cell through specialized uptake mechanisms (Schalk et al., 2012). A report from Barghouthi et al. (1989) showed that amonabactin is necessary for iron acquisition from Fe-transferrin in *A. hydrophila*. Further study showed that *amoA* was the key gene in the seven-gene cluster involved in the biosynthesis of amonabactin in *A. hydrophila* 495A2 (Barghouthi et al., 1991). To determine whether amonabactin was required for NE-induced growth promotion of *A. hydrophila* NJ-35 in the iron-restricted medium, we inactivated the *amoA* gene. The data here showed that whether in the presence or absence of NE, the *amoA* mutant showed a significant reduction in growth when compared with wildtype, indicating that amonabactin plays a crucial role in bacterial growth in the restrictive environment of serum-SAPI medium. However, the addition of NE significantly stimulated the growth of both wild-type and *amoA* mutant strains, suggesting that the NE-mediated iron acquisition from transferrin in iron-restricted medium is independent of the ability to synthesize amonabactin. The precise mechanism(s) by which NE modulates iron uptake from iron-Tf remain to be determined. Although, we speculate that NE-mediated iron uptake from iron-Tf might be due to NE acting directly in a siderophore-like manner, we could not exclude the possibility that NE-Tf interactions were releasing iron for subsequent uptake by bacterial iron acquisition systems. Future work will be carried out to elucidate this.

The process of siderophore-mediated iron acquisition from transferrin is energy-dependent. The active transport of iron-siderophore compounds across the outer membrane is energized by a complex of proteins called the TonB energy transduction system (Postle and Larsen, 2007). *A. hydrophila* possesses three TonB systems, similar to *Vibrio vulnificus* (Kustusch et al., 2012). To discover which TonB system was required in NE-mediated iron acquisition of *A. hydrophila*, we individually inactivated the three TonB systems. We found that all three *tonB* mutants exhibited lower growth yields compared with the wild-type strain in SAPI medium containing serum, indicating that all three TonB systems were involved in iron acquisition from Fe-transferrin in serum. However, in the presence of NE, the *tonB2* mutant demonstrated no growth promotion, although this stimulatory effect was observed for both the *tonB1* and *tonB3* mutants. This result indicates that the TonB2 system may play an important role in NE-induced iron acquisition from Fe-transferrin in serum, and TonB2-dependent growth promotion might be important for *A. hydrophila* infection in the host under stress. Moreover, the findings showed that both the Δ*tonB2-1* and Δ*tonB2-2* strains could not respond to NE in serum-containing medium, indicating that TonB2-1 and TonB2-2 are absolutely required for the function of the TonB2 system in *A. hydrophila*. The observation that NE-mediated growth promotion of *A. hydrophila* is TonB-dependent strongly suggests that a specific TonB-dependent outer membrane receptor might be involved in the transport of iron from transferrin via norepinephrine. Further studies are necessary to demonstrate which receptors are needed for NE-induced growth promotion of *A. hydrophila*.

In conclusion, our results demonstrate that the stress hormones NE, Epi, and Dopa stimulate bacterial growth and increase biofilm formation and cell adhesion ability in *A. hydrophila*. These results partially explain why stress increases the risk of *A. hydrophila* infection in fish. We also provide evidence to suggest that NE-induced growth promotion correlates with iron uptake from transferrin facilitated by norepinephrine. Although, the mechanisms of communication between bacteria and their hosts are not well understood, the modulation of bacterial growth and virulence by stress hormones brings a new perspective to infectious disease processes induced by bacteria.

## Author contributions

YL and YD conceived the study and drafted the paper. YD and JL performed most of the experiments described in the manuscript. HD, NW, and FA helped with the experiments. CL provided valuable suggestions of the manuscript. All authors read and approved the final manuscript.

### Conflict of interest statement

The authors declare that the research was conducted in the absence of any commercial or financial relationships that could be construed as a potential conflict of interest.
