# Supplementary material for: Catecholamine-Stimulated Growth of Aeromonas hydrophila Requires the TonB2 Energy Transduction System but Is Independent of the Amonabactin Siderophore
Source: Front Cell Infect Microbiol. 2016 Dec 12;6:183. doi: 10.3389/fcimb.2016.00183 (PMC5149522; doi:10.3389/fcimb.2016.00183)
Supplement: Supplementary file 1 [file Image1.PDF]

Supplementary Material

Catecholamine-stimulated Growth of *Aeromonas hydrophila* Requires the TonB2 Energy Transduction System but is Independent of the Amonabactin Siderophore

Yuhao Dong[1], Jin Liu[1], Maoda Pang[1,2], Hechao Du[1], Nannan Wang[1], Furqan Awan[1], Chengping Lu[1], Yongjie Liu[1*]

[1]College of Veterinary Medicine, Nanjing Agricultural University, Nanjing, China

[2] Key Lab of Food Quality and Safety of Jiangsu Province-State Key Laboratory Breeding Base, Institute of Food Safety, Jiangsu Academy of Agricultural Sciences, Nanjing, China.

*** Correspondence:**

Corresponding author

liuyongjie@njau.edu.cn

Supplementary Figures

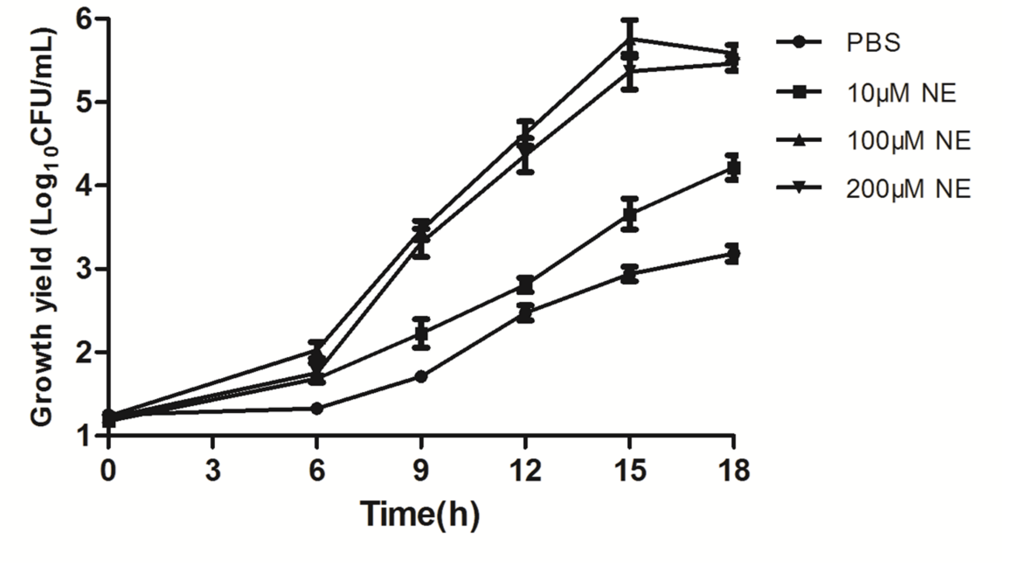

FIGURE S1. Effect of NE supplementation on *A. hydrophila* growth. *A. hydrophila* NJ-35 was grown in serum-supplemented SAPI medium in the presence of 10, 100, 200μM NE. The growth yield is expressed as Log_{10}CFU/ml, and results are expressed as the means ± SEM of three independent replicates.
